# Characteristics of Mammographic Breast Density and Associated Factors for Chinese Women: Results from an Automated Measurement

**DOI:** 10.1155/2019/4910854

**Published:** 2019-03-19

**Authors:** Tong Li, Lichen Tang, Ziba Gandomkar, Rob Heard, Claudia Mello-Thoms, Qin Xiao, Yajia Gu, Genhong Di, Carolyn Nickson, Zhimin Shao, Patrick Brennan

**Affiliations:** ^1^Medical Radiation Sciences, Faculty of Health Sciences, The University of Sydney, Cumberland Campus, 75 East Street, Lidcombe, NSW, Australia; ^2^Department of Breast Surgery, Key Laboratory of Breast Cancer in Shanghai, Fudan University Shanghai Cancer Center, 270 Dongan Road, Shanghai, China; ^3^Behaviour and Social Sciences, Faculty of Health Sciences, The University of Sydney, Cumberland Campus, 75 East Street, Lidcombe, NSW, Australia; ^4^Department of Radiology, Key Laboratory of Breast Cancer in Shanghai, Fudan University Shanghai Cancer Center, 270 Dongan Road, Shanghai, China; ^5^Centre for Epidemiology and Biostatistics, Melbourne School of Population and Global Health, University of Melbourne, 207 Bouverie Street, Melbourne, VIC 3010, Australia

## Abstract

**Background:**

Characteristics of mammographic density for Chinese women are understudied. This study aims to identify factors associated with mammographic density in China using a quantitative method.

**Methods:**

Mammographic density was measured for a total of 1071 (84 with and 987 without breast cancer) women using an automatic algorithm AutoDensity. Pearson tests examined relationships between density and continuous variables and t-tests compared differences of mean density values between groupings of categorical variables. Linear models were built using multiple regression.

**Results:**

Percentage density and dense area were positively associated with each other for cancer-free (r=0.487, p<0.001) and cancer groups (r=0.446, p<0.001), respectively. For women without breast cancer, weight and BMI (p<0.001) were found to be negatively associated (r=-0.237, r=-0.272) with percentage density whereas they were found to be positively associated (r=0.110, r=0.099) with dense area; age at mammography was found to be associated with percentage density (r=-0.202, p<0.001) and dense area (r=-0.086, p<0.001) but did not add any prediction within multivariate models; lower percentage density was found within women with secondary education background or below compared to women with tertiary education. For women with breast cancer, percentage density demonstrated similar relationships with that of cancer-free women whilst breast area was the only factor associated with dense area (r=0.739, p<0.001).

**Conclusion:**

This is the first time that mammographic density was measured by a quantitative method for women in China and identified associations should be useful to health policy makers who are responsible for introducing effective models of breast cancer prevention and diagnosis.

## 1. Introduction 

Breast cancer is the most commonly diagnosed neoplasm amongst women in China and it is one of the leading causes of cancer death in females [1]. Mammographic density, describing the amount of fibrous and glandular tissue within the breasts, is consistently demonstrated to be an important risk factor for breast cancer. Women with highest density were shown to have 2 to 6 times higher risk in developing breast cancer compared to those with the lowest [2]. Mammographic density is also associated with an elevated risk of masking tumours, which lowers the sensitivity of mammography [3] and therefore identifies women who may benefit from additional imaging such as breast ultrasound or magnetic resonance imaging [4]. Digital breast tomosynthesis might also be recommended [5].

Well-confirmed factors associated with higher density include younger age, lower body mass index (BMI), premenopausal status, nulliparity, late age at first delivery, a smaller number of live births, and family history of breast cancer [6]. However, current knowledge around density data is largely based on women from westernised countries and the characteristics of mammographic density for women in China are under studied [7]. From limited data that are available, Chinese mammographic density was shown to be positively associated with earlier age at menarche, premenopausal status, smaller number of children, later age at first delivery, and personal history of benign breast disease [8, 9]. Also, larger breast size was found to be negatively associated with density amongst premenopausal women in China [10].

Even though the previously mentioned studies investigated Chinese mammographic density, the associations predominantly focused on reproductive agents. In addition, all previous studies used the qualitative method of Breast Imaging Reporting and Data System (BI-RADS) classifications. Despite being the most commonly used assessment approach of mammographic density in both clinical settings and screening programs in China and many other countries [11, 12], the BI-RADS classification has been shown to suffer limited reproducibility with wide inter- (kappa = 0.02-0.77) and intrareader (kappa = 0.32-0.88) variations [13]. This subjectivity has the potential to result in inconsistent breast cancer risk prediction and unnecessary discrepancies in decision-making for density assessment [14]. As a consequence, automated methods using mathematical and physical principles have been designed to promote objective and consistent assessment of mammographic density.

The aim of the current work is to identify predictive factors of mammographic density for both Chinese women with and without breast cancer using a quantitative algorithm. Two density metrics will be considered, percentage density (PD) and dense area (DA) measures, and the impact of each metric on various associations will be explored.

## 2. Materials and Methods

### 2.1. Study Design and Population

This was a retrospective cross-sectional study. A total of 1000 women without breast cancer were recruited from the Breast Cancer Screening Program (BCSP) organized by Fudan University Shanghai Cancer Center (FUSCC) from March 2015 to June 2016. Another 100 women who had a pathologically confirmed diagnosis of breast cancer (ductal carcinoma in situ included) were randomly selected by Excel RAND function from the clinical environment at FUSCC during the same time period.

Ethical approval was obtained from the Human Research Ethics Committee of the University of Sydney (Project number: 2014/768) and the Institutional Review Board of Fudan University Shanghai Cancer Center (Project number 1503144-11). All data that came from FUSCC database were deidentified in this retrospective study and informed consent was waived.

### 2.2. Data Collection

Women's characteristics were obtained from the registration form and the discharge summary contained within the health record for each woman with breast cancer and through a BCSP questionnaire for breast cancer-free women. All the information for women was deidentified, with dedicated study IDs used to link mammograms and other data.

Details on height, weight, age at menarche, age at menopause, age at first delivery, and duration of breastfeeding were collected as continuous variables. Age at mammography was calculated by the assessment date and date of birth.

Ethnicities other than Han Chinese were classified into a single non-Han grouping and level of education was coded into a dichotomous variable in order to increase statistical power since these two variables with more than two groupings resulted in very uneven and low numbers in certain groups. Geographic location was also coded as a categorical variable with two groupings (Shanghai and other locations) since the program was conducted in Shanghai and consequently most of the participants came from Shanghai. Menopause status, parity history, number of children, breastfeeding history, personal history of breast cancer, family history of breast cancer, degree of consanguinity, smoking history, and history of alcohol consumption were also classified into two groupings which were specific to each variable detailed in the results.

All of the factors of interest mentioned above were collected for women without breast cancer; however ethnicity, smoking, alcohol history, level of education, and geographic location were unavailable for women with cancer since these details were not recorded on admission to FUSCC.

### 2.3. Image Acquisition

Mammograms taken closest in time to the cancer diagnosis and to the questionnaire completion were obtained for women with and without breast cancer, respectively. For all women, craniocaudal projection of both sides of breasts (where available) was accessed and these mammograms were acquired by Mammomat Inspiration (Siemens; Erlangen, Germany) or Selenia (Hologic, Inc., Bedford, MA, USA) units.

### 2.4. Mammographic Density Measurement

**Figure 1:**
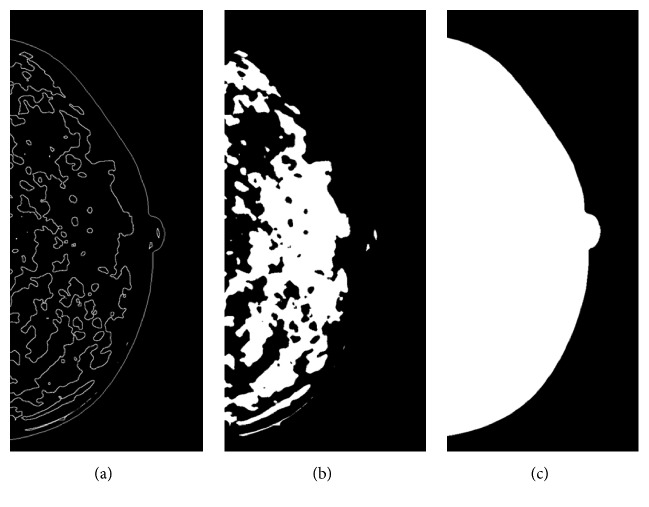
*Output from AutoDensity algorithm*. (a) White line delineates the edge of the breast and the edge of dense tissue. (b) Mask of dense issue within the breast. (c) Mask of area of the breast.

Mammographic density was measured by a fully automatic algorithm AutoDensity version 1.7, which identifies both areas of dense tissue (dense area) and of breast tissue (breast area) in mammograms and then classifies percentage mammographic density. This algorithm, which has been validated elsewhere [15], automatically finds an optimal threshold for each mammogram independently from any other images in a data set, in order to segment the breast from the background within a mammogram and outline the dense tissue within the breast (Figure 1(a)). Both the dense area (Figure 1(b)) and breast area (Figure 1(c)) are highlighted and the resultant PD was produced by dividing the dense area (number of pixels) by the breast area (number of pixels) and expressing in a percentage. Mammograms of both left and right breasts for each woman were assessed and the average value of both sides was used for all the statistical analyses. This algorithm was provided to the affiliation of the corresponding author in September 2016.

### 2.5. Statistical Analysis

The data derived from both the screening program (cancer-free women) and clinical settings (cancer women) were subjected to two types of statistical analysis: univariable and multivariable analysis. Women with and without cancer were analysed as separate groups, because the variable sets available for each group differed slightly.

The relationship between PD and continuous variables was assessed using the Pearson correlation coefficient (*r*). Difference of mean values of PD was compared between the groupings of each dichotomous variable using t-tests.

To identify key factors associated with PD, linear model building was performed using stepwise multiple regression adopting the significant variables from Pearson tests and t-tests except those restricted to women with specific conditions (for example, age at menopause was restricted to postmenopausal women only, so this variable was not used in the model building). Residuals of the PD were examined to check for assumptions of linear models by using regression scatterplots and histograms. R-squared statistics were used to assess the goodness of fit of the models.

All of the statistical tests performed for PD were repeated for DA.

SPSS (IBM SPSS statistics for windows, version 22.0) statistical package was used for all statistical analyses, and two-tailed tests of significance were employed using a significance level of 0.05.

## 3. Results

### 3.1. Characteristics of Participants

After excluding cases with unilateral images, a total of 1071 (84 with and 987 without breast cancer) women were finally selected for statistical analysis.  Table 1 shows the characteristics for both groups of women.

**Figure 2:**
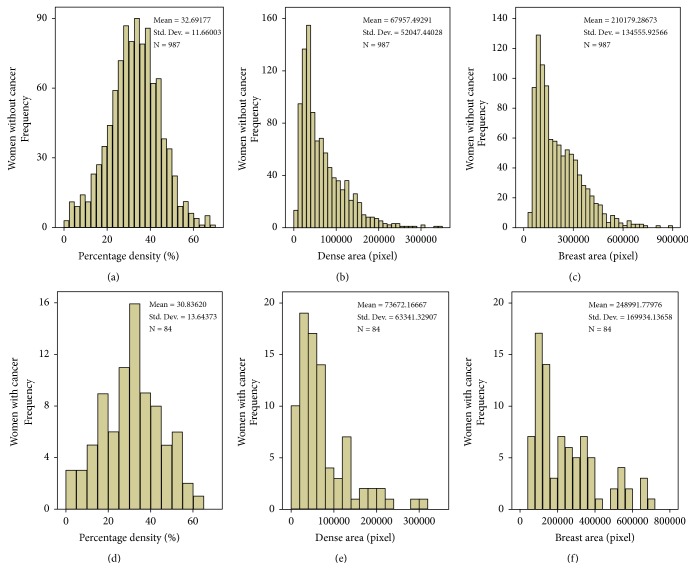
*Distribution of mammographic features*. (a) Percentage mammographic density (%) of women without breast cancer. (b) Area of dense tissue (pixel) in mammograms of women without breast cancer. (c) Area of breast tissue (pixel) in mammograms of women without breast cancer. (d) Percentage mammographic density (%) of women with breast cancer. (e) Area of dense tissue (pixel) in mammograms of women with breast cancer. (f) Area of breast tissue (pixel) in mammograms of women with breast cancer.

Figure 2 depicts the distribution of PD, DA, and breast area from AutoDensity algorithm for both cancer and cancer-free women.

### 3.2. Association between PD and DA

PD and DA were positively correlated for cancer-free women (r = 0.487, p < 0.001) and for women with cancer (r = 0.446, p < 0.001), respectively.

### 3.3. Determinants of Mammographic Density

The output from the Pearson and t-tests for both PD and DA are shown in Table 2 for both cancer and cancer-free women.

#### 3.3.1. Women without Breast Cancer

Age at mammography (r = -0.202), weight (r = -0.237), BMI (r = -0.272), and age at menarche (r = -0.078) were significantly and negatively associated (p < 0.001) with PD. Lower PD (p < 0.001) was found within postmenopausal women and women with secondary education background or below compared to premenopausal women and women with tertiary education.

DA was found to be positively associated with breast area (r = 0.790, p < 0.001), body weight (r = 0.110, p < 0.001), and BMI (r = 0.099, p = 0.002). Negative associations were shown between DA and age at mammography (r = -0.086, p = 0.007) and age at menarche (r = -0.080, p = 0.012). DA was also found to be lower in women with a history of nulliparity (p = 0.014) and lack of breastfeeding (p= 0.002) compared to women without such histories.

#### 3.3.2. Women with Breast Cancer

Negative associations were found between PD and age at mammography (r = -0.451, p < 0.001), weight (r = -0.495, p < 0.001), BMI (r = -0.520, p < 0.001), and age at menopause (r = -0.290, p = 0.046). Reduced PD was also found in women with postmenopausal compared with premenopausal status (p < 0.001).

Within this group of women, breast area was positively associated with DA (r = 0.739, p < 0.001).

### 3.4. Linear Models

Linear models were built for both PD and DA for each of the two groups of women (where for menopause status, 0 = premenopausal and 1 = postmenopausal, and for education level, 0 = secondary and below and 1 = tertiary). The equations of the 4 most-effective models (I-IV) are presented as follows and the residuals of these models were all normally distributed. The details of individual coefficients are in Supplementary Tables S1-S4.

- PD (cancer-free) = 54.99 - 1.00*∗*BMI - 4.42*∗*Menopause status + 2.19*∗*Education level. This model predicted 12.13% of the variation in PD (F = 45.21, p < 0.001).
- DA (cancer-free) = 49650.58 - 2251.99*∗*BMI - 7921.84*∗*Menopause status + 5582.45*∗*Education level + 0.32*∗*Breast area. This model successfully predicted 65.06% of DA variation for women without breast cancer (F = 457.17, p < 0.001).
- PD (cancer) = 97.16 - 1.80*∗*BMI - 0.43*∗*Age. BMI and age can account for 38.82% of the variation in PD (F = 25.70, p < 0.001).
- DA (cancer) = 0.28*∗*Breast area. This model with only one predictor predicted 54.56% of the variation in DA for cancer women (F = 98.48, p < 0.001).

## 4. Discussion

This study, for the very first time, identified a number of factors associated with mammographic density for women both with and without breast cancer in China by employing a fully automatic algorithm AutoDensity. Two measures provided by this algorithm were used to assess mammographic density in our study: PD and DA, which we found were moderately correlated with each other. Previous studies that compared the differences of prediction of breast cancer risk between these two measures suggested that the cancer risk associated with DA was stronger than or as strong as that with PD [16, 17]. By combining the effects of the constituting measures [18], PD delivers limited information regarding the absolute amount of dense tissues which are potentially at risk of undergoing a malignant transformation [19]. To illustrate, when a certain amount of dense tissue is measured within a small breast, a relatively higher percentage will be provided, compared to the identical amount of target tissue measured within a large breast. However, even though it may therefore be argued that PD is not an appropriate measure of choice in etiologic research, it is very commonly used to present mammographic density since it is an easily applicable and practicable prognostic factor of breast cancer risk [20]. This might partially result from the fact that percentage density appears to be less affected by technical issues such as the degree of breast compression [16].

Determinants for both measures were demonstrated within our study; however predictors were not consistent for PD and DA. An example of this inconsistency is breast area, which accounted for more than half of the DA variation for both cancer and cancer-free women, whereas it appeared to have no impact on PD. This was particularly noticeable for women diagnosed with cancer since breast area is the only factor arising from univariate analysis that was statistically significant.

Associations of body weight and BMI were dependent on which of the two measures were used. The negative associations of mammographic density with increasing BMI and increasing weight that have been shown for the percentage metric have been shown for several decades across many populations [21]. In contrast, DA was found to be positively associated with weight and BMI, which is not aligned with most of the westernised-based literature [17, 20, 21]. However a similar finding was shown in studies involving Chinese women living in westernised and developed countries [22, 23]. The alignment with our work suggests that the positive association (although not strong) between BMI/weight and DA might be unique to Chinese mammographic density. Nevertheless, this hypothesis will need further study to be proven or disproven because there is very limited literature on this topic that our results can be compared to (see Introduction). The question, however, remains of why would relationships appear in the opposite directions in our work focusing on Chinese women depending on whether PD or DA is used as the dependent variable.

Another important finding was that women in tertiary education appeared to have denser breasts compared to those women with lower level of education. This finding is consistent with previous studies from Europe and North American with a focus on Caucasian women [24, 25]. To our knowledge, this is the first time the relationship between mammographic density and education for women in China has been shown. This could have important future implications since Chinese people are increasingly keen to undergo tertiary education, for example, the graduation rate from tertiary education institutions increased by three times over the last two decades [26]. Other socioeconomic factors, e.g., employment, household income, home ownership, urbanisation/ruralisation, and social class, associated with higher education levels, may also impact on this relationship [25], but this was not investigated in our study.

Despite displaying a negative association with mammographic density within the univariate analysis, age at mammography did not add any prediction beyond other variables within the multivariate model for PD or DA in women without breast cancer. This is inconsistent with previous work based on either Chinese [10, 22] or other populations [17, 27] and may suggest a characteristic only relevant to women in our study and not applicable to the general Chinese female population. Another explanation is that the contributions of other elements within the multivariate models had a much greater impact than that of age at mammography or that age has already been modelled by proxy through menopause, which is highly correlated to age in the optimal model (r = 0.762, p < 0.001).

The available data around relationships between density and smoking history and alcohol consumption for populations other than Chinese are inconsistent. Some studies found a positive association with alcohol consumption [28] and a negative association with smoking history [29], whereas others showed no associations [30]. We also failed to identify any association with these two lifestyle factors, which is consistent with two previous work focusing on Chinese women [8, 10], but may be partially explained by the low number of women in our study with a positive smoking or alcohol intake history (less than 15%), and information only being available for women without breast cancer. With regard to ethnic variations, this is the first time that density was studied between women of Han origin and non-Han origin in China and no associations were shown, which was different to that seen for ethnic variations in other populations [31]. This finding however should be treated with some degree of caution since all the ethnic minority groups at data collection were categorized as non-Han origin in order to increase statistical power, since the total number of women in this study belonging to specific ethnic minorities was very low (<2%). This aggregation could be obscuring minority-specific observations, an issue that needs to be addressed in further work.

This study used a fully automatic algorithm to measure mammographic density for women in China. Even though, in the clinical and screening settings in China, the BI-RADS scheme is the most commonly used classification to assess density, this visual approach is relatively time-consuming and requires more workload from radiologists compared to quantitative computer aided methods [32]. Also, the reproducibility of BI-RADS classification is questionable due to the subjectivity of readers involved with density assessment [33]. Even though it is the first time that AutoDensity has been used for density assessment for Chinese women, it has been shown to be comparable to Cumulus, a globally employed semiautomatic algorithm, in terms of association with breast cancer risk and breast cancer screening outcomes in Australia [15]. This approach allowed important associations to be identified but also revealed that one must standardise and understand better the metric being used. In addition, AutoDensity is a breast area-based algorithm instead of a volume-based algorithm. AutoDensity is therefore based on the projected area, rather than the volume of breast tissues, and consequently finds a threshold between dense and nondense areas. Therefore the thickness of the breast is not taken into account during the AutoDensity measurement. This potential source of error in measurement is likely to attenuate the observed relationship between percentage density/dense area and potential determinants and risk of breast cancer.

Nevertheless, this study has a few limitations. As menopause was shown to be an important and contributing factor for Chinese mammographic density, different menopausal status might have important influences on the density values. However, we did not separate pre-, peri- and postmenopausal women in our study, which will be the focus of further work. Also, the small sample size of women in the cancer group is noted. A larger sample of women with cancer may have revealed further relationships, and future studies seeking to recruit larger samples of women diagnosed with cancer are recommended. Besides, we acknowledge that the lack of follow-up period after mammograms in our study may be a challenge. But due to the fact that the follow-up period was not a standard process of BCSP, we were unable to collect these data. This could mean that the cancer-free women may contain missed breast cancer and thus increased values of both PD and DA. Finally, we did not provide a comparative analysis using both quantitative (i.e., AutoDensity) and qualitative (i.e., BI-RADS) measurements because BI-RADS scales were not routinely reported in the BCSP. But this could be a focus of further work.

In conclusion, this study for the first time in China demonstrated important determinants of mammographic density in AutoDensity-generated PD and DA values. Differences between the two density metrics emphasise the importance of understanding better what each metric represents for both women with and without breast cancer and ensuring that approaches are standardised. We believe our findings should be valuable to health policy makers who are responsible for introducing effective models of breast cancer prevention and diagnosis.

## Figures and Tables

**Table 1:** Characteristics of women with and without breast cancer.

Variables	Women without breast cancer		Women with breast cancer	
Continuous	N^a^	M±SD^b^	N	M±SD
Percentage mammographic density (%)	987	32.69±11.66	84	30.84±13.64
Dense area (pixels)	987	67957.49±52047.44	84	73672.17±63341.33
Breast area (pixels)	987	210179.29±134555.93	84	248991.78±169934.14
Age at mammography (years)	987	48.94±9.49	84	52.99±11.19
Height (cm)^c^	987	160.63±4.74	84	159.73±3.80
Weight (kg)^d^	987	57.84±7.90	84	61.75±9.01
BMI (kg/m^2^)^e^	987	22.40±2.81	84	24.19±3.34
Age at menarche (years)	987	14.22±1.88	84	15.20±1.62
Age at menopause (years)^f^	370	50.39±3.80	48	50.23±3.81
Age at first delivery^g^	926	27.84±3.48	84	25.54±3.46
Duration of breastfeeding^h^	762	7.21±4.14	77	15.92±14.03

Categorical	N	%^i^	N	%

Menopause status				
Pre-menopausal	617	62.50	36	42.86
Post-menopausal	370	37.50	48	57.14
Parity status				
Nulliparous	61	6.18	0	0.00
Parous	926	93.82	84	100.00
Number of children^g^				
=1	853	92.10	50	59.52
>1	73	7.90	34	40.48
Breastfeeding history				
No	225	22.80	7	8.33
Yes	762	77.20	77	91.67
Personal history of breast cancer				
No	977	98.99	83	98.81
Yes	10	1.01	1	1.19
Family history of breast cancer				
No	915	92.71	76	90.48
Yes	72	7.29	8	9.52
Degree of consanguinity^j^				
1st degree	47	65.28	7	87.50
2nd degree	25	34.72	1	12.50
Ethnicity				
Non-Han origin	11	1.11	N/A^k^	N/A
Han origin	976	98.89	N/A	N/A
Smoking history				
No	967	97.97	N/A	N/A
Yes	20	2.03	N/A	N/A
Alcohol consumption				
No	864	87.54	N/A	N/A
Yes	123	12.46	N/A	N/A
Level of education				
Secondary and below	176	17.83	N/A	N/A
Tertiary	811	82.17	N/A	N/A
Geographic location				
Shanghai	800	81.05	N/A	N/A
Others	187	18.95	N/A	N/A

^a^Number of cases.

^b^Mean ± standard deviation for continuous variables.

^c^Height range for women without breast cancer: 146.00-180.00; height range for women with cancer: 15.00-171.00.

^d^Weight range for women without breast cancer: 38.00-90.00; weight range for women with cancer: 42.00-89.00.

^e^Calculated by weight (kg)/[height (m)]^2^, weight range (cancer-free).

^f^Restricted to postmenopausal women.

^g^Restricted to parous women.

^h^Restricted to women with breastfeeding history.

^i^Percentage of cases for categorical variables.

^j^Restricted to women with family history.

^k^Not available.

**Table 2:** Output from univariate analysis of PD and DA for women with and without breast cancer.

Variables	Women without breast cancer				Women with breast cancer			
	Percentage density		Dense area		Percentage density		Dense area	
Continuous	r^a^	P^b^	r	P	r	P	r	P
Breast area (pixels)	-0.043	0.174	0.790	<0.001	-0.142	0.197	0.739	<0.001
Age at mammography (years)	-0.202	<0.001	-0.086	0.007	-0.451	<0.001	-0.011	0.920
Height (cm)	0.030	0.352	0.034	0.283	-0.028	0.803	0.041	0.708
Weight (kg)	-0.237	<0.001	0.110	<0.001	-0.495	<0.001	-0.075	0.498
BMI (kg/m^2^)^c^	-0.272	<0.001	0.099	0.002	-0.520	<0.001	-0.088	0.425
Age at menarche (years)	-0.078	0.014	-0.080	0.012	-0.084	0.447	0.104	0.344
Age at menopause (years)^d^	0.007	0.887	-0.003	0.950	-0.290	0.046	-0.225	0.124
Age at first delivery^e^	-0.026	0.437	-0.014	0.678	0.157	0.155	-0.021	0.852
Duration of breastfeeding^f^	-0.068	0.059	0.058	0.110	-0.173	0.133	-0.081	0.486

Categorical	M±SD^g^	P	M±SD	P	M±SD	P	M±SD	P

Menopause status								
Pre-menopausal	34.85±10.89	<0.001	72178.87±53101.24	<0.001	37.06±11.36	<0.001	78641.39±60101.65	0.364
Post-menopausal	29.09±12.01		60918.05±49515.81		26.17±13.44		69945325±66046.94	
Number of children^e^								
=1	32.74±11.58	0.250	69656.06±52925.99	0.101	33.02±13.06	0.075	72208.90±60232.08	0.954
>1	31.09±13.69		59848.41±47664.33		27.62±14.04		75824.03±68528.80	
Breastfeeding history								
No	31.76±11.15	0.173	58874.45±41826.50	0.002	39.18±14.20	0.091	95541.71±98622.02	0.380
Yes	32.97±11.80		70639.49±54436.41		30.08±13.43		71684.03±59713.81	
Personal history of breast cancer								
No	32.71±11.66	0.724	67935.83±52101.27	0.939	30.80±13.72	0.832	72544.88±62872.93	0.132
Yes	31.40±1.33		70073.65±48958.06					
Family history of breast cancer								
No	32.58±11.76	0.278	68018.35±52313.70	0.991	30.74±13.43	0.844	73065.19±63672.86	0.644
Yes	34.13±10.27		67184.01±48874.61		31.75±16.57		79438.44±63969.64	
Degree of consanguinity^h^								
1st degree	33.31±9.55	0.354	67363.28±46429.23	0.857	29.14±16.02	0.269	83744.14±67831.43	0.659
2nd degree	35.68±11.56		66846.98±54173.46		N/A^i^		N/A	
Parity status								
Nulliparous	34.00±9.96	0.367	53909.78±41139.40	0.014		N/A		N/A
Parous	32.61±11.76		68882.88±52573.07					
Ethnicity								
Non-Han origin	37.25±3.81	0.192	98537.64±65684.04	0.255		N/A		N/A
Han origin	32.64±11.65		67612.84±51812.92					
Smoking history								
No	32.72±11.63	0.625	67877.39±51861.52	0.902		N/A		N/A
Yes	31.43±13.30		71830.55±61786.61					
Alcohol consumption								
No	32.84±11.61	0.291	68481.84±52155.77	0.365		N/A		N/A
Yes	31.65±11.99		64274.29±51338.71					
Level of education								
Secondary and below	28.67±12.19	<0.001	61590.39±47918.12	0.040		N/A		N/A
Tertiary	33.56±11.36		69339.25±52827.48					
Geographic location								
Shanghai	32.50±11.50	0.276	67946.45±51558.11	0.861		N/A		N/A
Others	33.53±12.30		68004.75±54233.54					

^a^Pearson's correlation coefficient for continuous variables.

^b^P values from Pearson and t-tests for continuous and categorical variables, respectively.

^c^Calculated by weight (kg)/[height (m)]^2^.

^d^Restricted to postmenopausal women.

^e^Restricted to parous women.

^f^Restricted to women with breastfeeding history.

^g^Mean ± standard deviation.

^h^Restricted to women with family history.

^i^Not applicable due to insufficient number/unavailable data.

## Data Availability

The images and dataset used to support the findings of this study are restricted by the Human Research Ethics Committee of the University of Sydney and the Institutional Review Board of Fudan University Shanghai Cancer Center in order to protect patient privacy and confidentiality. Data may be available to researchers who meet the criteria for access to confidential data by contacting the corresponding author.

## Supplementary Materials

Supplementary Material Supplementary Table S1: linear models of predictors of percentage mammographic density for women without breast cancer. Supplementary Table S2: linear models of predictors of dense area for women without breast cancer. Supplementary Table S3: linear models of predictors of percentage mammographic density for women with breast cancer. Supplementary Table S4: linear models of predictors of dense area for women with breast cancer.
