# Supplementary material for: Characteristics of Mammographic Breast Density and Associated Factors for Chinese Women: Results from an Automated Measurement
Source: J Oncol. 2019 Mar 19;2019:4910854. doi: 10.1155/2019/4910854 (PMC6444251; doi:10.1155/2019/4910854)
Supplement: Supplementary Material — Supplementary Table S1: linear models of predictors of percentage mammographic density for women without breast cancer. Supplementary Table S2: linear models of predictors of dense area for women without breast cancer. Supplementary Table S3: linear models of predictors of percentage mammographic density for women with breast cancer. Supplementary Table S4: linear models of predictors of dense area for women with breast cancer. [file 4910854.f1.docx]

| **Supplementary table S1: Linear models of predictors of percentage mammographic density for women without breast cancer** | | | | | | | | | | | | |
| --- | --- | --- | --- | --- | --- | --- | --- | --- | --- | --- | --- | --- |
| Model | | Unstandardized Coefficients B | 95% Confidence Interval for B | | Standard Error for B | Standardized Coefficients Beta | P value | R | R^2^ | R^2^ change | ANOVA | |
|  |  |  | Lower bound | Upper bound |  |  |  |  |  |  | F | P value |
| 1 | Constant | 57.9103 | 52.2827 | 63.5380 | 2.8678 |  | <0.001 | 0.2720 | 0.0739 | 0.0739 | 78.5511 | <0.001 |
|  | BMI | -1.1257 | -1.3749 | -0.8765 | 0.1270 | -0.2718 | <0.001 |  |  |  |  |  |
| 2 | Constant | 57.3789 | 51.8782 | 62.8795 | 2.8031 |  | <0.001 | 0.3420 | 0.1167 | 0.0429 | 65.0287 | <0.001 |
|  | BMI | -1.0179 | -1.2633 | -0.7725 | 0.1251 | -0.2457 | <0.001 |  |  |  |  |  |
|  | Menopause status | -5.0247 | -6.4512 | -3.5981 | 0.7269 | -0.2087 | <0.001 |  |  |  |  |  |
| 3^a^ | Constant | 54.9967 | 49.1262 | 60.8671 | 2.9915 |  | <0.001 | 0.3480 | 0.1213 | 0.0045 | 45.2135 | <0.001 |
|  | BMI | -1.0021 | -1.2474 | -0.7567 | 0.1250 | -0.2419 | <0.001 |  |  |  |  |  |
|  | Menopause status | -4.4200 | -5.9384 | -2.9016 | 0.7738 | -0.1836 | <0.001 |  |  |  |  |  |
|  | Education level | 2.1917 | 0.2774 | 4.1059 | 0.9755 | 0.0720 | 0.0249 |  |  |  |  |  |
| ^a^ Model I in the main text | | | | | | | | | | | | |

| **Supplementary table S2: Linear models of predictors of dense area for women without breast cancer** | | | | | | | | | | | | |
| --- | --- | --- | --- | --- | --- | --- | --- | --- | --- | --- | --- | --- |
| Model | | Unstandardized Coefficients B | 95% Confidence Interval for B | | Standard Error for B | Standardized Coefficients Beta | P value | R | R^2^ | R^2^ change | ANOVA | |
|  |  |  | Lower bound | Upper bound |  |  |  |  |  |  | F | P value |
| 1 | Constant | 3707.0527 | 9.2900 | 7404.8154 | 1884.3300 |  | 0.0490 | 0.7900 | 0.6246 | 0.6246 | 1638.6398 | <0.001 |
|  | Breast area | 0.3057 | 0.2909 | 0.3205 | 0.0076 | 0.7903 | <0.001 |  |  |  |  |  |
| 2 | Constant | 56827.5991 | 41127.3583 | 72527.8400 | 8000.6211 |  | <0.001 | 0.8010 | 0.6415 | 0.0170 | 880.4860 | <0.001 |
|  | Breast area | 0.3206 | 0.3055 | 0.3357 | 0.0077 | 0.8288 | <0.001 |  |  |  |  |  |
|  | BMI | -2511.0261 | -3233.2396 | -1788.8126 | 368.0298 | -0.1358 | <0.001 |  |  |  |  |  |
| 3 | Constant | 55698.4651 | 40150.7714 | 71246.1588 | 7922.8753 |  | <0.001 | 0.8060 | 0.6492 | 0.0076 | 606.2572 | <0.001 |
|  | Breast area | 0.3192 | 0.3043 | 0.3342 | 0.0076 | 0.8253 | <0.001 |  |  |  |  |  |
|  | BMI | -2289.4186 | -3010.4407 | -1568.3966 | 367.4222 | -0.1238 | <0.001 |  |  |  |  |  |
|  | Menopause status | -9464.1439 | -13482.5157 | -5445.7722 | 2047.7029 | -0.0881 | <0.001 |  |  |  |  |  |
| 4^a^ | Constant | 49650.5805 | 33065.1263 | 66236.0346 | 8451.6921 |  | <0.001 | 0.8070 | 0.6506 | 0.0015 | 457.1725 | <0.001 |
|  | Breast area | 0.3194 | 0.3045 | 0.3344 | 0.0076 | 0.8258 | <0.001 |  |  |  |  |  |
|  | BMI | -2251.9898 | -2972.7747 | -1531.2048 | 367.3009 | -0.1218 | <0.001 |  |  |  |  |  |
|  | Menopause status | -7921.8374 | -12201.3916 | -3642.2831 | 2180.7950 | -0.0737 | <0.001 |  |  |  |  |  |
|  | Education level | 5582.4523 | 191.3059 | 10973.5987 | 2747.2452 | 0.0411 | 0.0420 |  |  |  |  |  |
| ^a^ Model II in the main text | | | | | | | | | | | | |

| **Supplementary table S3: Linear models of predictors of percentage mammographic density for women with breast cancer** | | | | | | | | | | | | |
| --- | --- | --- | --- | --- | --- | --- | --- | --- | --- | --- | --- | --- |
| Model | | Unstandardized Coefficients B | 95% Confidence Interval for B | | Standard Error for B | Standardized Coefficients Beta | P value | R | R^2^ | R^2^ change | ANOVA | |
|  |  |  | Lower bound | Upper bound |  |  |  |  |  |  | F | P value |
| 1 | Constant | 82.2348 | 63.5239 | 100.9457 | 9.4057 |  | <0.001 | 0.5200 | 0.2706 | 0.2706 | 30.4248 | <0.001 |
|  | BMI | -2.1251 | -2.8915 | -1.3587 | 0.3853 | -0.5202 | <0.001 |  |  |  |  |  |
| 2^a^ | Constant | 97.1620 | 78.3448 | 115.9791 | 9.4573 |  | <0.001 | 0.6230 | 0.3882 | 0.1176 | 25.6960 | <0.001 |
|  | BMI | -1.8028 | -2.5277 | -1.0780 | 0.3643 | -0.4413 | <0.001 |  |  |  |  |  |
|  | Age | -0.4288 | -0.6451 | -0.2125 | 0.1087 | -0.3518 | <0.001 |  |  |  |  |  |
| ^a^ Model III in the main text | | | | | | | | | | | | |

| **Supplementary table S4: Linear models of predictors of dense area for women with breast cancer** | | | | | | | | | | | | |
| --- | --- | --- | --- | --- | --- | --- | --- | --- | --- | --- | --- | --- |
| Model | | Unstandardized Coefficients B | 95% Confidence Interval for B | | Standard Error for B | Standardized Coefficients Beta | P value | R | R^2^ | R^2^ change | ANOVA | |
|  |  |  | Lower bound | Upper bound |  |  |  |  |  |  | F | P value |
| 1^a^ | Constant | 5115.8963 | -11491.4018 | 21723.1944 | 8348.2347 |  | 0.5420 | 0.7390 | 0.5456 | 0.5456 | 98.4760 | <0.001 |
|  | Breast area | 0.2753 | 0.2201 | 0.3305 | 0.0277 | 0.7387 | <0.001 |  |  |  |  |  |
| ^a^ Model IV in the main text | | | | | | | | | | | | |
